# An early post-operative ACTH suppression test can safely predict short- and long-term remission after surgery of Cushing’s disease

**DOI:** 10.1007/s11102-018-0902-6

**Published:** 2018-07-23

**Authors:** Erik Uvelius, Peter Höglund, Stig Valdemarsson, Peter Siesjö

**Affiliations:** 1 Neurosurgery, Department of Clinical Sciences Lund, Skåne University Hospital, Lund University, EA-Blocket Plan 3, 221 85 Lund, Sweden; 2 Laboratory Medicine, Department of Clinical Chemistry & Pharmacology, Lund University, Lund, Sweden; 3 Department of Clinical Sciences, Skåne University Hospital, Lund University, Lund, Sweden

**Keywords:** ACTH-secreting pituitary adenoma, Cushing disease, Pituitary adenoma, Pituitary neoplasms/surgery, Transphenoidal surgery, Recurrence, Treatment outcome

## Abstract

**Purpose:**

The present study evaluates the usefulness of an ACTH suppression test shortly after surgery, and to determine optimal cut-off values of included laboratory analyses, in predicting short- and long-term remission after surgery of Cushing’s disease.

**Methods:**

A 48 h suppression test with betamethasone 2 mg/day applied after 45 transphenoidal adenomectomies in 28 patients was evaluated. Receiver operating characteristic (ROC)-curves were created for the included assays: plasma cortisol, plasma adrenocorticotropic hormone (ACTH) and urinary free cortisol (UFC). Plasma levels of cortisol and ACTH were measured both at 24 and 48 h. Youden’s index was used to determine cut-off with the highest sensitivity and specificity in predicting short- (3 months) and long-term (5 years or longer) remission. The area under curve (AUC) illustrated the clinical accuracy of the different assays.

**Results:**

Plasma cortisol after 24 h with betamethasone was most accurate in predicting both short- and long-term remission. 3 months remission with cut-off 107 nmol/L: sensitivity 0.85, specificity 0.94, positive predictive value (PPV) 0.96 and AUC 0.92 (95% CI 0.85–1). 5 years remission with cut-off 49 nmol/L: sensitivity: 0.94, specificity 0.93, PPV 0.88, AUC 0.98 (95% CI 0.95–1). Analyses of ACTH or UFC did not improve diagnostic accuracy.

**Conclusions:**

A 48 h, 2 mg/day betamethasone suppression test after transphenoidal surgery of Cushing’s disease could predict short- and long-term remission with a high accuracy. Suppression of plasma cortisol after 24 h with betamethasone to values excluding Cushings disease in the diagnostic setting yielded the highest accuracy in predicting long-term remission.

## Introduction

Cushing’s disease is caused by hypersecretion of adrenocorticotropic hormone (ACTH) by a pituitary adenoma resulting in hypercortisolism [1]. Surgical adenomectomy is the first line of treatment [2]. Postoperative remission is reported in 43–95% of cases depending of factors such as adenoma size, finding of pituitary adenoma on preoperative MRI and surgeons experience [3–12]. However, there is no consensus on what laboratory assays and biochemical thresholds should be used in determining or predicting remission over time [6, 9]. Resolution of symptoms, urinary free cortisol (UFC), plasma cortisol nadir, plasma ACTH and low-dose dexamethasone suppression test (LDDST) are used in various constellations [9]. At our center withdrawal of per operative glucocorticoid substitution is followed by a suppression test with betamethasone, 2 mg/day during 48 h, initiated on postoperative day 2 for an early evaluation of the effect of surgery in patients with Cushing’s disease. Like dexamethasone, betamethasone is a potent synthetic glucocorticoid, which does not interfere in biochemical assays measuring plasma or urine cortisol. Potent synthetic glucocorticoids cause a significant feedback inhibition on ACTH, which could be expected to result in very low plasma cortisol levels if all adenoma tissue is removed, while adenoma residues left behind should still be less responsive causing continued ACTH production and elevated cortisol levels.

The rationale for exchange of peroperative glucocorticoid substitution to the present test protocol is twofold: (1) to achieve an early evaluation of the effect of surgery and (2) to avoid the risk for acute hypocortisolism without necessitating a high level of clinical surveillance [13]. After completion of the test, patients are deemed in remission and given continued glucocorticoid substitution or considered uncured and thus not in need of substitution. The present retrospective study was undertaken to clarify the ability of this early suppression test to predict short- and long-term remission after surgery of Cushing’s disease and to determine the optimal cut-off values of the laboratory analyses included in the test.

## Materials and methods

### Patients

Data was retrospectively collected from medical records of patients who presented with Cushing’s disease between November 1998 and December 2011. All surgical procedures were performed at the Department of Neurosurgery, Skåne University Hospital, which is a referral center for 1.7 million inhabitants and the only center in the region for pituitary surgery. The study period was selected to allow at least a 5 years follow-up period. During the recruitment period 47 patients in all were diagnosed with Cushing’s disease. After exclusions of pediatric patients, adult patients unfit for surgery due to comorbidity or lack of follow up and patients not completely tested according to the protocol, 28 patients remained eligible for evaluation of the presented protocol. 4 patients had died at the end of the final minimum follow-up period (December 2016) and were included without consent. The remaining 24 patients were asked to participate. All agreed and were included after giving written consent. The final number of surgical procedures to be evaluated was 45, performed in 28 patients as primary procedures or reoperations.

### Preoperative work-up

Consultant pituitary endocrinologists confirmed Cushing’s disease by a combination of clinical examination, dexamethasone suppression tests and UFC. Inferior petrosal sinus sampling (IPSS) was used in 21 cases (75%) before primary surgery. All patients had sellar MRIs. Findings on MRI before the primary procedure and before repeat surgery are listed in Table 1. Regarding primary procedures 79% (22/28) were performed with preoperative MRI showing adenoma. Including reoperations, the figure was 71% (32/45).

**Table 1:** Patient demographics

	Primary surgery (n = 28)	Reoperations (n = 17)	p-value
Age, years, median (range)	49.5 (23–77)	42 (28–64)	0.62^a^
Female gender, n (%)	20 (71)	8 (47)	0.11^b^
Surgical technique, n (%)			
Microsurgery	7 (25)	2 (12)	0.29^b^
Endoscopy	21 (75)	15 (88)	
Adenoma category, n (%)			
Not visible on MRI	6 (21)	6 (35)	0.67^b^
Micro-adenoma	11 (39.5)	8 (47)	0.49^b^
Macro-adenoma	11 (39.5)	2 (12)	0.12^b^
Missing	0 (0)	1 (6)	0.38^b^
Procedures/patient, n (%)			
1 Procedure	15 (54)		
2 Procedures	9 (32)		
3 Procedures	4 (14)		

Basic demographics of patients divided by primary surgery and reoperation where no significant differences were seen between the groups

^a^Two sample *t* test

^b^Fisher exact test

### Surgery and perioperative care

All surgical procedures were performed via the transphenoidal route. Microsurgical techniques were used from 1998 to 2004, from 2004 the fully endoscopic transphenoidal technique was used. Intravenous and per oral glucocorticoid substitution was administered pre, per and postoperatively as described in Table 2.

**Table 2:** Protocol of glucocorticoid substitution and hormone analyses used in the 48 h, 2 mg/day betamethasone suppression test

	Pre operative	Day of surgery	Post operative			
	Day 1	Day 0	Day 1	Day 2	Day 3	Day 4
Glucocorticoid substitution	Hydrocortisone 100 mg im 23.00	Hydrocortisone 50 + 50 + 50 mg iv	Hydrocortisone 50 + 25 + 25 mg iv	Betamethasone 1 + 0.5 + 0.5 mg p.o.	Betamethasone 1 + 0.5 + 0.5 mg p.o.	Hydrocortisone 20 + 10 + 10 mg p.o. (or no supplements^a^)
Biochemical analyses	–	–	–	Plasma cortisol + Plasma ACTH	Plasma cortisol + plasma ACTH UFC-sampling	Plasma cortisol + plasma ACTH

Glucocorticoid substitution was given three times daily (8 AM, 2 PM and 8 PM). Samples for biochemical analyses were taken prior to the morning dose (8 AM). The success of surgery was evaluated on postoperative day 4. If the patient was in remission continuous substitution with hydrocortisone was prescribed

^a^If surgical success was not achieved substitution was discontinued

### 48 h, 2 mg/day betamethasone suppression test

The peroperative glucocorticoid substitution with hydrocortisone was ended 12 h before commencement of the suppression test and 36 h before the first evaluation of serum cortisol during betamethasone suppression (day 3). This allowed us to avoid the risk of acute hypocortisolism and at the same time evaluate the effect of surgery and decide on the need of further glucocorticoid substitution. Betamethasone was administered orally (1 mg at 8 AM, 0.5 mg at 2 PM and 0.5 mg at 8 PM) during postoperative day 2 and 3. Morning levels (8 AM) of plasma cortisol and plasma ACTH were measured before commencement of the test and after 24 and 48 h with betamethasone (postoperative day 2, 3 and 4). Urine was collected for 24 h-UFC-analysis during the third postoperative day. The test protocol is presented in Table 2 and bare resemblance to the 48 h LDDST described by Liddle in 1960 [14].

In the present protocol, betamethasone was used as this was available and recognizable by staff in the neurosurgical ward where the patients were treated after surgery. Betamethasone and dexamethasone are both synthetic glucocorticoids with long effect duration, similar anti-inflammatory effect and negligible mineralocorticoid effect. Equipotency is reported at 1–1.25 mg dexamethasone per 1 mg betamethasone [15, 16]. We see no pharmacologic reason not to use our protocol with similar doses of dexamethasone.

### Hormone analyses and chemical assays

Included chemical analyses were plasma levels of cortisol and ACTH as well as UFC collected from days after surgery and four times during follow-up (3, 12 months, 5 years after surgery and at last known follow-up). The analyses were made at different clinical chemical laboratories with different assays. Reference ranges were known for all results. As the chemical methods varied over time, plasma cortisol and UFC levels were recalculated by equations provided by the laboratory to fit the current reference ranges used at the main laboratory (Skåne University Hospital) where plasma cortisol (reference range 07.00–10.00: 133–537 nmol/L)was analyzed via an accredited one-step competition assay (COBAS® 8000, Roche Diagnostics GmbH, Mannheim, Germany) and UFC (reference range: 30–170 nmol/24 h) was analyzed via an accredited mass spectrometry method (QTRAP® 5500 LC/MS/MS, SCIEX, Framingham, MA, USA). Different methods were used to measure plasma ACTH but no conversion equations were available. Therefore, the ACTH results were divided into five groups based on relation to the reference range of the method used in analogy with Invitti et al. [17]. Group 1: values below 50% of the lower reference limit, group 2: between 50% below lower reference limit and the lower reference limit, group 3: values within lower half of reference range; group 4: values within upper half of reference range, group 5: values above upper reference limit.

### Postoperative follow-up and definitions of remission and recurrence

Plasma levels of morning cortisol and ACTH along with UFC and/or overnight 1 mg LDDST were analyzed according to endocrinologist’s preference at follow-up 3 months after surgery and then yearly as well as at time of suspected recurrence. Remission was defined as decreasing clinical signs and symptoms of hypercortisolism in combination with laboratory test (UFC, morning plasma cortisol and ACTH, and/or an overnight 1 mg LDDST with significant plasma cortisol suppression). Unclear cases were always evaluated with overnight 1 mg LDDST to determine remission. Furthermore, patients unable to quit postoperative cortisol supplements were considered to be in continuous remission. As to recurrence, signs and symptoms in combination with incomplete suppression in overnight 1 mg LDDST, a flat 24 h plasma cortisol curve and/or UFC above reference range measured twice, defined recurrence. During the period studied, the outcome of biochemical testing, clinical signs and symptoms formed the basis for the endocrinologist’s compiled evaluation to determine remission or recurrence. These evaluations were reassessed by the authors based on the laboratory results and correlated well with the endocrinologist’s previous conclusions.

### Statistical methods

Statistical analyses were performed with the free statistical software R [18]. Receiver operating characteristic (ROC) curves illustrated sensitivity and specificity of postoperative measurements in determining immediate post-operative success and remission at later times (3, 12 months, 5 years after surgery and at last known follow-up). The Youden index was used to determine optimal cut-off values with highest sensitivity and specificity. In the analyses, sensitivity equals the test’s ability to correctly predict patients in remission whereas specificity in this setting is the tests ability to identify patients with later recurrence. Sensitivity and specificity were considered equally important. Area under the ROC curves (AUC) with 95% confidence interval were calculated to determine the predictive power of the laboratory analyses. AUC values between 0.7 and 0.9 indicate moderate accuracy whereas AUC values over 0.9 indicate high accuracy. ROC curves were drawn for primary surgeries, reoperations and the both combined. Two sample *t* test and Fisher exact test were used in determining differences between primary operations and reoperations. Linear and logistic univariate and multivariable regressions determined pre- and peri-operative factors influencing surgical outcome. Analyses were made to determine factors influencing remission, and thus might affect the cut-off values of the 48 h, 2 mg/day betamethasone suppression test. Factors with p < 0.1 in univariate analyses were included in multivariable regression analysis. The following factors were analyzed: Age-group (age in years < 38,39–44, 45–58 and > 59), primary procedure or reoperation, gender, surgical technique (microsurgery or endoscopic surgery), the use of IPSS, occurrence of complications after surgery as well as adenoma size (non-visible, micro- or macro-adenoma). p-Values < 0.05 were considered significant. Specific ROC-curves were to be plotted for the subgroups showing significance in multivariable regression analyses. These analyses were intended to clarify if subgroups were in need of specific biochemical cut-off values of the chemical assays.

## Results

### Patients

The majority of patients were females (71%) and the median age 49.5 years. There were no significant differences between patients undergoing a primary procedure or reoperation regarding age, gender or surgical technique. Two patients were diagnosed with pituitary carcinoma and 11 of the 28 patients had a macro-adenoma (39%). Pathology reports showed finding of adenoma after primary procedures and reoperations in 93 and 87%, respectively. All adenomas were ACTH positive in immunohistochemical stainings. Two cases of ACTH-producing carcinomas were confirmed by pathologists. Patient demographics are given in Table 1.

Surgical complications were rare. Reoperation did not carry a significantly higher risk of complications. One patient experienced persistent, > 12 months, postoperative diabetes insipidus. Except for need of glucocorticoid substitution after successful surgery, no other case of reduced pituitary function was found according to endocrinological evaluation. No adrenal crises were observed.

### Surgical results

Twelve of the 28 patients (43%) showed 5 years and longer long-term remission at last follow-up (median 116 months) after one surgical intervention. Three patients were not considered in remission after primary surgery but never candidates for further surgical intervention. 13 out of the 28 patients included underwent a total of 17 reoperations (9 patients had 1 reoperation and 4 patients had 2 reoperations) due to residual disease or recurrence. Four patients showed long term remission after a second or third procedure, thus, in total, long-term remission (5 years or longer) was seen in 16 patients (57%) after surgical treatment, including patients cured after repeated surgery. All 16 remained in remission at last known follow up (median 110 months). Four patients, not considered cured according to direct post-operative assessment, fulfilled remission criteria at 3 months. However, only one of these patients were in remission 5 years after surgery. None of these four patients had malignant disease but two had a slightly increased proliferative activity and one had dural invasion. Two patients who, by endocrinologist assessment, indicated immediate postoperative remission showed recurrence after 3 months. Median follow-up time, between first surgery and either last known follow-up, recurrence or death, was 71 months (range 3–206 months). All recurrences occurred within 54 months of surgery. Nine (20%) procedures were initially deemed successful but patients later had recurrences.

### Factors influencing remission

In univariate analyzes of both 3 months-remission and 5 years remission, gender, the use of IPSS and age-group in the upper quartile showed p < 0.1 and were included in multivariable analysis. In multivariable regression analysis, male gender was the only factor significantly associated with lower remission rates in either short or long-term follow-up, p = 0.002 and 0.047 respectively.

### Results of the 48 h, 2 mg/day betamethasone suppression test

**Fig. 1:**
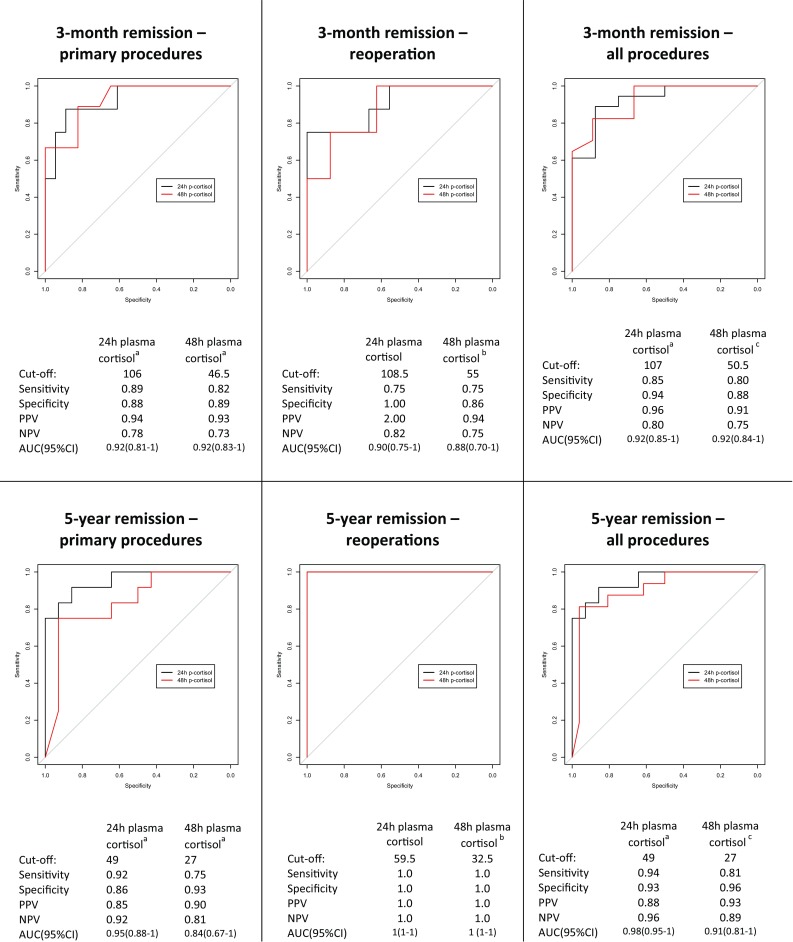
ROC curves with optimal cut-off values and corresponding sensitivity, specificity and AUC for the two best predictors of remission. The first column visualizes ROC-curves for primary procedures (n = 28) and the ability of plasma cortisol levels after 24 and 48 h with betamethasone to predict remission after 3 months and after 5 years. The second column visualizes corresponding ROC-curves for reoperations (n = 17). The third column visualizes corresponding ROC-curves for all procedures (primary intervention and reoperations combined, n = 45). The data indicates that the optimal cut-off values are not influenced by primary surgery or surgery after failure or recurrence. Plasma cortisol is presented in nmol/L. ^a^2 missing values, ^b^1 missing value, ^c^3 missing values

In summary, the best predictors in determining remission were plasma cortisol after 24 and 48 h with betamethasone. ROC-curves with sensitivity, specificity at the optimal plasma cortisol cut-off and AUC in predicting 3 months and 5 years remission are presented in Fig. 1. The cortisol cut off values for 12 months prediction (data not shown) were similar to those for 3 months. The cut-off values after reoperations as well as primary procedures and reoperations combined are shown in Fig. 1. Plasma ACTH and UFC analyses are not shown as ROC-curves since they all had lower sensitivity, specificity and lower accuracy or had larger number of missing values. As there were cases were patients showed remission first 3 months after surgery and long-term follow-up was considered more important than 12 months remission the following analyses were focused on 3 months and 5 years remission. No recurrences were seen later than 54 months after surgery, thus values of last follow-up equal 5 years remission values.

Sensitivity and specificity of selected different plasma cortisol cut-offs at 24 and 48 h in the present betamethasone suppression test versus outcome at 3 months and 5 years are given in Table 3. 140 nmol/L was originally reported to be the normal suppression level of a LDDST and 83 nmol/L is the cut-off values previously used by Chen et al. [19] in predicting long-term remission. In our material, these thresholds lack in specificity. The suppression cut-off used in diagnosis of Cushing’s disease, 50 nmol/L, was close to the cut-off level with the highest sensitivity and specificity in our series (49 nmol/L). Finally, 25 nmol/L exemplifies that a more stringent suppression level will cause a low level of true positives and larger number of false negatives, or a high specificity at the expense of sensitivity.

**Table 3:** Sensitivity and specificity of selected plasma cortisol cut-offs applied to the 48 h 2 mg/day betamethasone suppression test

	24 h Plasma cortisol		48 h Plasma cortisol	
	3 months	5 years + last follow-up*	3 months	5 years + last follow-up^a^
Cut-off 140 nmol/L				
Sensitivity	0.94	1.00	1.00	1.00
Specificity	0.75	0.50	0.67	0.42
AUC (95% CI)	0.92 (0.81–1.0)	0.95 (0.88–1.0)	0.92 (0.83–1.0)	0.84 (0.67–1)
Cut-off 83 nmol/L				
Sensitivity	0.78	0.92	0.92	0.92
Specificity	0.88	0.71	0.50	0.50
AUC (95% CI)	0.92 (0.81–1.0)	0.95 (0.88–1.0)	0.92 (0.83–1.0)	0.84 (0.67–1)
Cut-off 50 nmol/L				
Sensitivity	0.67	0.92	0.82	0.83
Specificity	0.88	0.86	0.89	0.64
AUC (95% CI)	0.92 (0.81–1)	0.95 (0.88–1)	0.92 (0.83–1.0)	0.84 (0.67–1)
Cut-off 25 nmol/L				
Sensitivity	0.33	0.50	0.47	0.58
Specificity	1.00	1.00	1.00	0.93
AUC (95% CI)	0.92 (0.81–1)	0.95 (0.88–1)	0.92 (0.83–1.0)	0.84 (0.67–1)

See “Results” section for selected cut-offs. AUC illustrates the predictive power of each assay and is not affected by changing cut-off values

^a^No recurrences were seen later than 54 months after surgery, thus values of last follow-up equal 5 years remission values

## Discussion

Applied on 45 pituitary surgical procedures in 28 patients with Cushing’s disease, our results indicate that a postoperative betamethasone suppression test is a safe and accurate way to predict both short- and long-term remission after transphenoidal surgery. The usefulness of the betamethasone test presented is underlined by the high accuracy that was achieved even though the heterogeneous material included a large proportion of macro-adenomas and cases of pituitary cancer. Cut-off values were equal regardless of primary or repeated surgery. Furthermore, considering no pre- or peri-operative factor, except for gender, significantly influenced remission and the assay used to measure plasma cortisol has no gender specific reference ranges, we consider the cut-off values universal in regard of preoperative factors.

Despite that the test was performed as a 48 h suppression test, the best accuracy in predicting long-term remission was assessment of plasma cortisol after 24 h with betamethasone. Application of this test, with a 49 nmol/L cut-off, yields a sensitivity of 0.94 with a specificity of 0.93. AUC 0.98 with a narrow 95% confidence interval, also indicates a high diagnostic accuracy. A test result with suppression below 49 nmol/L will correctly predict 5 years remission in 88% of cases. Applying this method on the present series of patients and using it as the sole predictor of future remission, suppression of plasma cortisol below cut-off was seen after 17 procedures. Two of these patients had recurrences, while, 5 years after surgery 15 patients (88%) were still in remission. This could be compared with procedures after which suppression did not reach cut-off value (n = 26). In the latter group, 8 patients were considered in remission by endocrinologist’s compiled assessment after surgery. None of the eight were in remission 5 years after surgery.

It has been shown that the LDDST, in the healthy individual, suppresses plasma cortisol below 140 nmol/L [20]. In our series of patients, a plasma cortisol cut-off at 140 nmol/L after betamethasone was a poor predictor of remission. High sensitivity as well as high specificity was only achieved with a plasma cortisol cut-off δ50 nmol/L (Table 3).

Even though dexamethasone suppressions tests are often used during follow-up of Cushing’s disease [9], we have found only two studies reporting results of early LDDST for prediction of remission after surgery. Chen et al. [19] report 174 patients recruited over a 20 years period showing a 93% chance of 5 years remission if suppression in an overnight LDDST was below 83 nmol/L. With reservation to methodological differences in cortisol assays, applying the cut-off 83 nmol/L to our patient series gave inferior specificity (Table 3). Atkinson et al. [21] retrospectively reviewed their series of 63 patients where LDDST was used during the first week after surgery in determining remission where 45 patients reached early remission. 10 patients later had recurrent disease. Suppression to undetectable levels was more common amongst patients still in remission (88.9%) versus patients with recurrences who suppressed below detectable levels in 50% of cases. However, these studies used different strategies to diagnose remission, highlighting the fact that different remission criteria are practiced. If incorporating undetectable levels of plasma cortisol as cut-off, or further lowering the plasma cortisol cut-off as is exemplified with cut-off 25 nmol/L in Table 3 we see enhanced specificity on the expense of sensitivity with increasing numbers of false negative results.

We use hydrocortisone substitution perioperatively and during the first days after surgery with a half-life of 1–2 h. Extending the betamethasone test over 48 h should secure against analytical interference from peri-operative hydrocortisone substitution. In addition, this protocol might also overcome the possibility of increased glucocorticoid metabolism as has recently been reported for dexamethasone [22]. With this in mind, we expected the results after 48 h with betamethasone to show the highest accuracy in predicting remission. However, the 24 h results seemed to yield slightly better predictions than the 48 h results. This could be by chance or the fact that little is known of how surgery affects the response of either normal pituitary or residual adenoma in postoperative suppression tests. Furthermore, the fact that the 24 h data seemed superior to the 48 h data argues against an interference from peroperatively administered hydrocortisone in the cortisol data. Thus, a 24 h test may well be used instead of the 48 h test making the test applicable also with shorter hospital stays.

Many institutions rely on plasma cortisol nadir in predicting remission. Low plasma cortisol nadir has showed to be highly correlated with remission, although with variable cut-off values as well as definitions of long-term remission [10, 23–30] Measuring plasma cortisol nadir requires withholding glucocorticoid substitution with the risk, although apparently minor [23, 24, 26, 28], of acute hypocortisolism. We have found no reports of serious adrenal crisis post-surgery in the literature, but we would expect withholding cortisol to be more labor intensive in retaining a high level of patient safety [13, 23, 27]. As we never pause substitution extensive monitoring is not needed to avoid adrenal crises.

One issue with any test used in determining remission after surgery of Cushing’s disease concern patients with delayed remission. The present study has not escaped this difficulty. 4 patients (14%) in our series experienced remission first at 3 months follow-up. Similar difficult to explain observations have been reported in between 5.6 and 20% of cases in previous studies [8, 31, 32], quite in line with our findings.

To conclude, a 48 h 2 mg/day betamethasone suppression test day 2 and 3 after transphenoidal surgery of Cushing’s disease could safely predict short- and long-term remission with high accuracy. Plasma cortisol after 24 h of suppression showed the best accuracy in predicting 5 years remission. Until consensus on remission criteria, it is still the endocrinologists combined assessment that defines remission. The present data supports that an early suppression test can be helpful in this assessment in predicting remission over time with high accuracy without the risk of adrenal crisis.
